# Bridging the Gap: A Pre- and Post-evaluation of an Online Skin of Colour Dermatology Lecture Series for Medical Students

**DOI:** 10.7759/cureus.96634

**Published:** 2025-11-11

**Authors:** Vaseharan Suntharan, Charlotte Wilson, Emma Amoafo, Dominique Dao, Ezigbo Iyamah, Marisa Taylor

**Affiliations:** 1 Dermatology, Epsom and St Helier NHS Trust, London, GBR; 2 Emergency Department, Epsom and St Helier NHS Trust, London, GBR; 3 Dermatology, Kingston and Richmond NHS Trust, London, GBR; 4 Internal Medicine, Morriston Hospital, Swansea, GBR; 5 Internal Medicine, Guy's and St Thomas' NHS Foundation Trust, London, GBR

**Keywords:** equity in dermatology education, equity in healthcare, health-care equity, medical education med ed learning classroom integrated, online dermatology, online teaching

## Abstract

Background

Medical students often report limited teaching on dermatology in skin of colour (SoC), which may contribute to lower confidence and risk of health inequalities.

Objective

The objective of this study is to evaluate a pilot student-led lecture series designed to bridge the gap in confidence between recognising dermatological conditions in fairer skin tones (Fitzpatrick I-III) and darker skin tones (Fitzpatrick IV-VI).

Methods

A nine-lecture online series was delivered to medical students. Students completed pre- and post-series surveys measuring self-reported confidence in diagnosing general dermatological (GenDerm) and SoC dermatological conditions on a five-point Likert scale. Changes in confidence scores were assessed with the Wilcoxon signed-rank test, and effect sizes were calculated. For dichotomised outcomes (high confidence ≥ quite confident), McNemar’s test with odds ratios (ORs) and 95% confidence intervals (CIs) was applied.

Results

A total of 44 students completed both surveys. Before the series, 88% (39/44) had not received formal SoC teaching; 30/44 (68.2%) felt more confident diagnosing in lighter vs darker skin. Most began the series with low confidence, particularly in SoC, where over half rated themselves 'not at all confident.' After the teaching, many moved into the somewhat confident or quite confident categories, and no students remained 'not at all confident.'

In GenDerm, confidence rose from a median of slightly confident to somewhat confident (Wilcoxon z = 4.13, p < 0.001, r = 0.69). In SoC, the improvement was greater, with the median shifting from not at all confident to somewhat confident (Wilcoxon z = 5.11, p < 0.001, r = 0.84). Both represent large effects, showing that the programme had a strong impact on student confidence. Although overall confidence rose sharply, fewer students crossed into the 'high confidence' category, reflecting the short duration of the programme. Support for including SoC teaching in the undergraduate curriculum was almost universal (>95%).

Conclusions

This pilot shows that a student-led lecture series was associated with improved student confidence in SoC. The aim was not to create experts in a short space of time, but to bring confidence with SoC more in line with confidence in GenDerm. By the end of the series, that gap had narrowed, suggesting that focused teaching can help to close the gap. Whilst these findings sit within Kirkpatrick levels 1 and 2 (reaction and self-reported learning), the series was associated with large improvements in student confidence. Larger, multi-centre studies using validated assessments are needed to see how this translates into diagnostic skills and patient care.

## Introduction

Racial and ethnic disparities in healthcare are well documented, with bias shown to affect clinical decision-making and patient outcomes [1-4]. Within dermatology, these disparities are compounded by inadequate training on conditions presenting in darker skin types. A survey reported that nearly half of U.S. dermatologists felt undertrained in diagnosing conditions in Black patients [5]. Although skin cancer is less common in individuals with darker skin tones, mortality rates are higher due to diagnostic delays [6].

Confidence among clinicians and students in diagnosing dermatological conditions also varies by skin tone. In one large-scale U.S. study, diagnostic accuracy for darker skin was significantly lower compared with lighter skin [7]. Smaller single-centre studies corroborate this, showing reduced diagnostic ability and confidence among medical students when presented with darker skin phototypes [8,9].

Educational resources have historically underrepresented SoC, with textbooks and training models predominantly depicting lighter skin [10-12]. This perpetuates uncertainty in practice and risks poorer outcomes for patients of colour. In the UK, dermatology teaching is often limited to short modules with minimal clinical exposure, exacerbating disparities [13].

To address this gap, we developed Bridging the Gap, a nine-lecture online series on SoC dermatology, with the aim of improving medical student confidence in recognising presentations in Fitzpatrick phototypes IV and above. For the purposes of this study, we refer to these as SoC presentations, whilst acknowledging that in practice such cases are part of GenDerm. The findings were initially presented as a poster at the British Association of Dermatologists Annual Meeting 2024, where feedback was received regarding the inclusion of statistical analysis, which has since been incorporated.

## Materials and methods

Study design and setting 

This educational intervention was organised by St George’s University Dermatology Society in collaboration with dermatology societies from Imperial College London, University College London, Queen Mary University of London, and King’s College London. The series was supported by Skin of Colour Training UK, the St George’s University Equity Champions programme, and the British Skin Foundation. The project was conceived and coordinated by the lead author (VS), then a fourth-year medical student at St George’s University, who led the design of the teaching programme, organisation across institutions, and delivery of the intervention in collaboration with faculty and student dermatology societies. 

Ethics 

This project was conducted in accordance with the St George’s Research Ethics Committee (SGREC) Standard Operating Procedure (JRESGOVSOP0054, Version 4.0, 20/05/2021). Under this SOP, studies that constitute educational interventions that do not involve patient participants, clinical data, or identifiable personal information are classified as exempt from requiring full SGREC review. As this study evaluated the impact of a dermatology lecture series on medical student confidence, it fell into the category of an educational intervention and was therefore exempt from formal ethics review. No SGREC application or approval number was required. Participation was voluntary, and completion of the surveys was taken as implied informed consent. 

Intervention 

Nine online lectures were delivered weekly between November 2023 and February 2024 (excluding the Christmas break) via Microsoft Teams (Microsoft Corporation, Redmond, WA). Sessions were delivered by consultant dermatologists, registrars and medical students. The titles of the lectures were as follows: skin care and acne in diverse skin types, common community (GP) presentations in diverse skin types, Afro-textured hair and colourism, paediatric dermatology in diverse skin types, skin cancer in diverse skin types, what to know about dermatology in African and Caribbean patients, rheumatological skin presentations in diverse skin types, South Asian skin presentations and vitiligo. 

A small attendance fee (three pounds per student) was donated in full to the British Skin Foundation. Lecture notes and supplementary materials were hosted on a dedicated website (www.diversedermatology.co.uk) to enhance accessibility and sustainability of the teaching. 

Participants 

Only medical students were included. For analysis, only those who attended four or more lectures and completed both pre- and post-series surveys were included (n = 44). The threshold of four sessions was chosen as it represented just under half of the lecture series, ensuring participants had sufficient exposure to the teaching to allow a meaningful evaluation of changes in confidence. 

Data collection

Pre- and post-lecture surveys were administered via Microsoft Forms (Microsoft Corporation, Redmond, WA) (Appendices). Surveys included questions on confidence in GenDerm and SoC, as well as perceptions of the importance of SoC teaching. Confidence was measured on a five-point Likert scale (1 = not at all confident, 2 = slightly confident, 3 = somewhat confident, 4 = quite confident, 5 = extremely confident), with ‘high confidence’ defined as ≥ four. 

For the survey, SoC dermatology was defined as conditions presenting in Fitzpatrick skin phototype IV or higher. We acknowledge that in practice, these conditions are part of GenDerm, but this separation was made to evaluate confidence specifically in darker skin types. The survey instrument was developed through a systematic process. Content validity was established through expert review by two consultant dermatologists, including one with formal qualifications in medical education (PGCert), who assessed the clinical relevance and educational appropriateness. Following expert review, the survey was piloted with 10 medical students to assess clarity and comprehensibility before final implementation. 

Statistical analysis 

Survey responses were summarised descriptively. Pre- and post-series confidence scores were compared using the Wilcoxon signed-rank test. For ease of interpretation, confidence ratings were also collapsed into two outcomes of ‘high confidence’ (≥‘quite confident’) versus all other responses. These two categories were assessed using McNemar’s test, with odds ratios (ORs) and 95% confidence intervals (CIs) calculated. Perceptions of the teaching, such as usefulness and the value of including skin of colour (SoC) in the curriculum, were reported as proportions with Wilson 95% CIs, which remain reliable even when responses were unanimous. Computational tools were used to generate the statistical calculations.

Post-hoc power calculations were undertaken using paired t-test approximations. The study was highly powered to detect the observed changes, with power exceeding 99% for both general dermatology (Cohen’s d = 0.81) and SoC (Cohen’s d = 1.34).

## Results

Baseline dermatology teaching 

**Figure 1:**
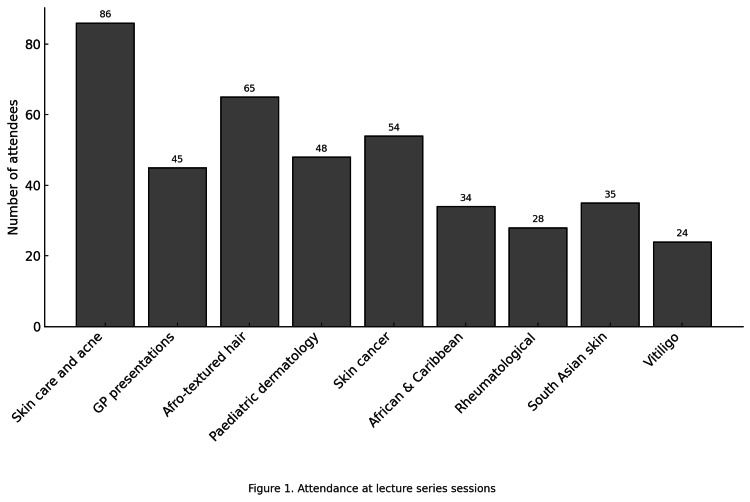
Attendance at lecture series sessions

Attendance data for each lecture are shown in Figure 1. The mean attendance across the nine lectures was 46.5 students. In total, 108 unique email addresses were recorded across the series, representing both undergraduate and postgraduate attendees. Attendance decreased as the series progressed, more so after the Christmas break.

Baseline confidence was low across both domains. In GenDerm, only 7% (3/44) of students reported high confidence (≥quite confident), whilst in SoC this was even lower at 2% (1/44). In addition, 68% (30/44) reported greater confidence in diagnosing conditions in lighter compared with darker skin. 

Perceptions of the importance of SoC teaching were high even before the series. All students (100%, 44/44) agreed that at least one lecture on diverse skin presentations should be included in every medical school curriculum, and almost all agreed that learning about dermatology in patients of colour is important. Table 1 and Table 2 provide numerical data for both pre- and post-lecture series.

**Table 1:** Pre-series self-reported confidence SoC, skin of colour

Confidence level	GenDerm n (%)	SoC n (%)
Not at all confident	34% (n = 15)	52% (n = 23)
Slightly confident	30% (n = 13)	32% (n = 14)
Somewhat confident	30% (n = 13)	14% (n = 6)
Quite confident	7% (n = 3)	2% (n = 1)
Extremely confident	0%	0%

**Table 2:** Post-series self-reported confidence (n = 44) SoC, skin of colour

Confidence level	GenDerm n (%)	SoC n (%)
Not at all confident	0 (0%)	0 (0%)
Slightly confident	9 (21%)	9 (21%)
Somewhat confident	19 (43%)	18 (41%)
Quite confident	15 (34%)	15 (34%)
Extremely confident	1 (2%)	2 (4%)

Confidence in GenDerm 

**Figure 2:**
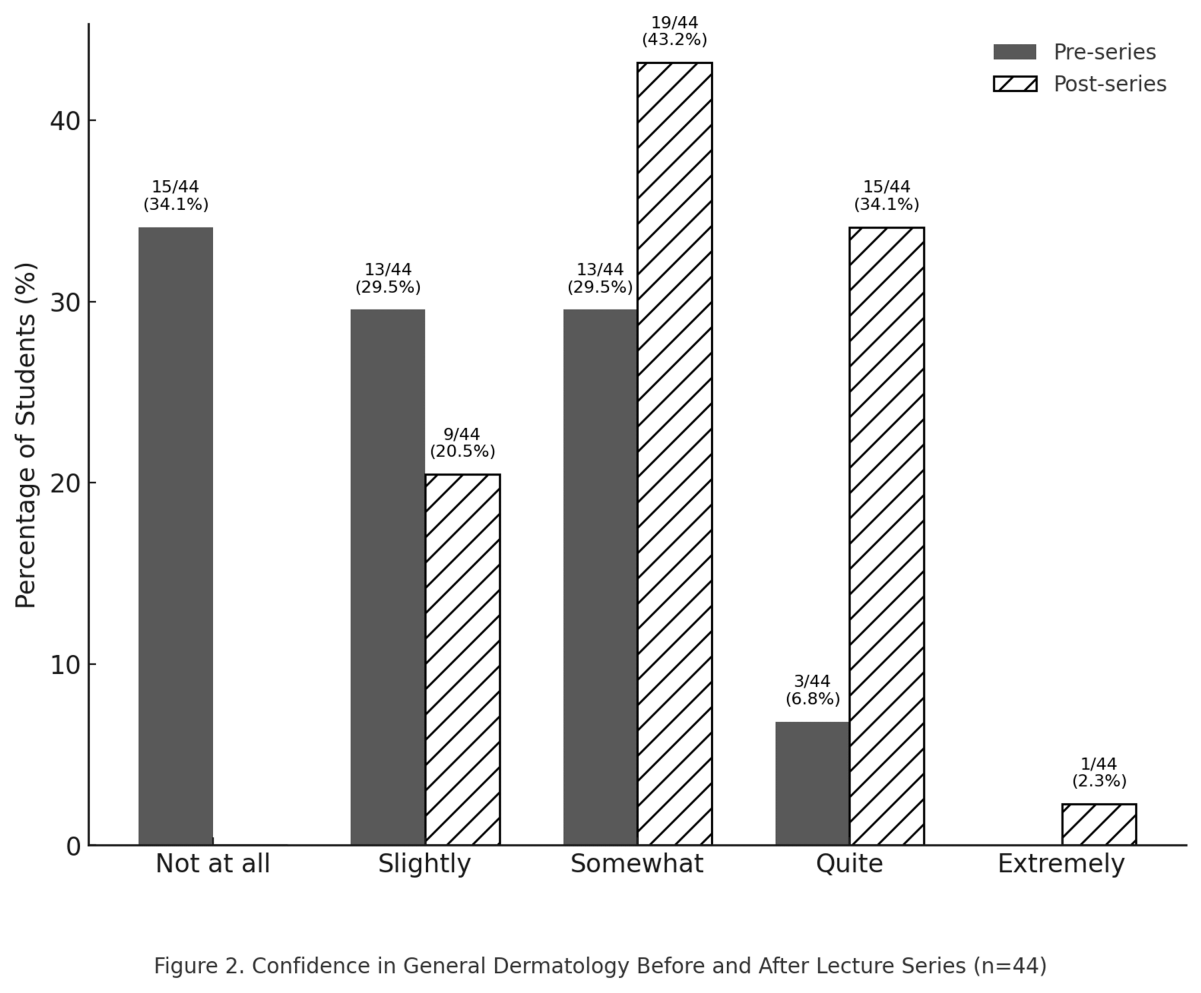
Comparison of student confidence in GenDerm before and after the lecture series (n = 44)

Before the series, most students described themselves as not at all or slightly confident, and only 7% (3/44) reported high confidence (≥quite confident). After the series, this increased to 36% (16/44). Median confidence rose from slightly confident to somewhat confident. This improvement was significant (Wilcoxon z = 4.13, p < 0.001, r = 0.69). Overall confidence improved across the scale, but only a few students moved into the highest confidence group. The data are illustrated in Figure 2.

Confidence in SoC 

**Figure 3:**
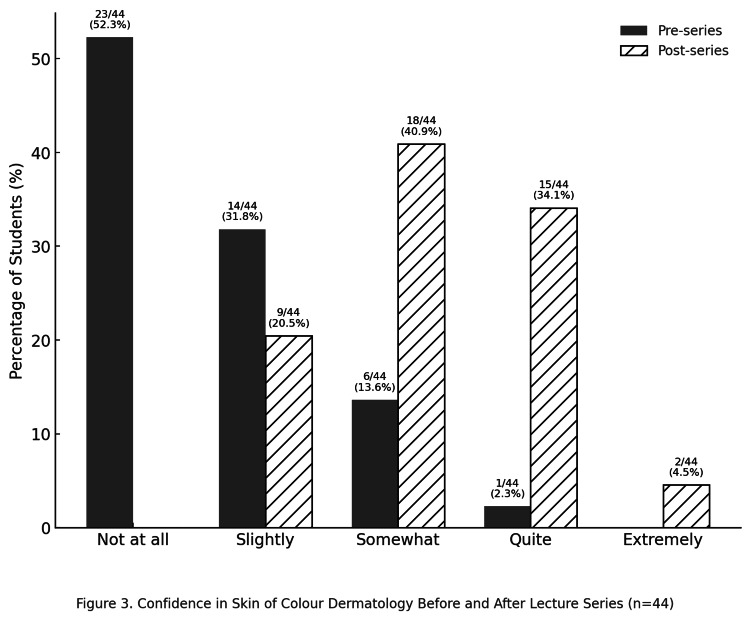
Comparison of student confidence in skin of colour (SoC) before and after the lecture series (n = 44)

At baseline, most students (52%, 23/44) described themselves as not at all confident, and only 2% (1/44) reported high confidence (≥quite confident). After the series, this rose to 39% (17/44). Median confidence increased from not at all confident to somewhat confident, a highly significant improvement (Wilcoxon z = 5.11, p < 0.001, r = 0.84). McNemar’s test showed a significant increase in the proportion of participants reporting high confidence (GenDerm: b = 15, c = 2, p = 0.002; SoC: b = 16, c = 0, p < 0.001). The data are illustrated in Figure 3.

Perceptions of the teaching 

**Figure 4:**
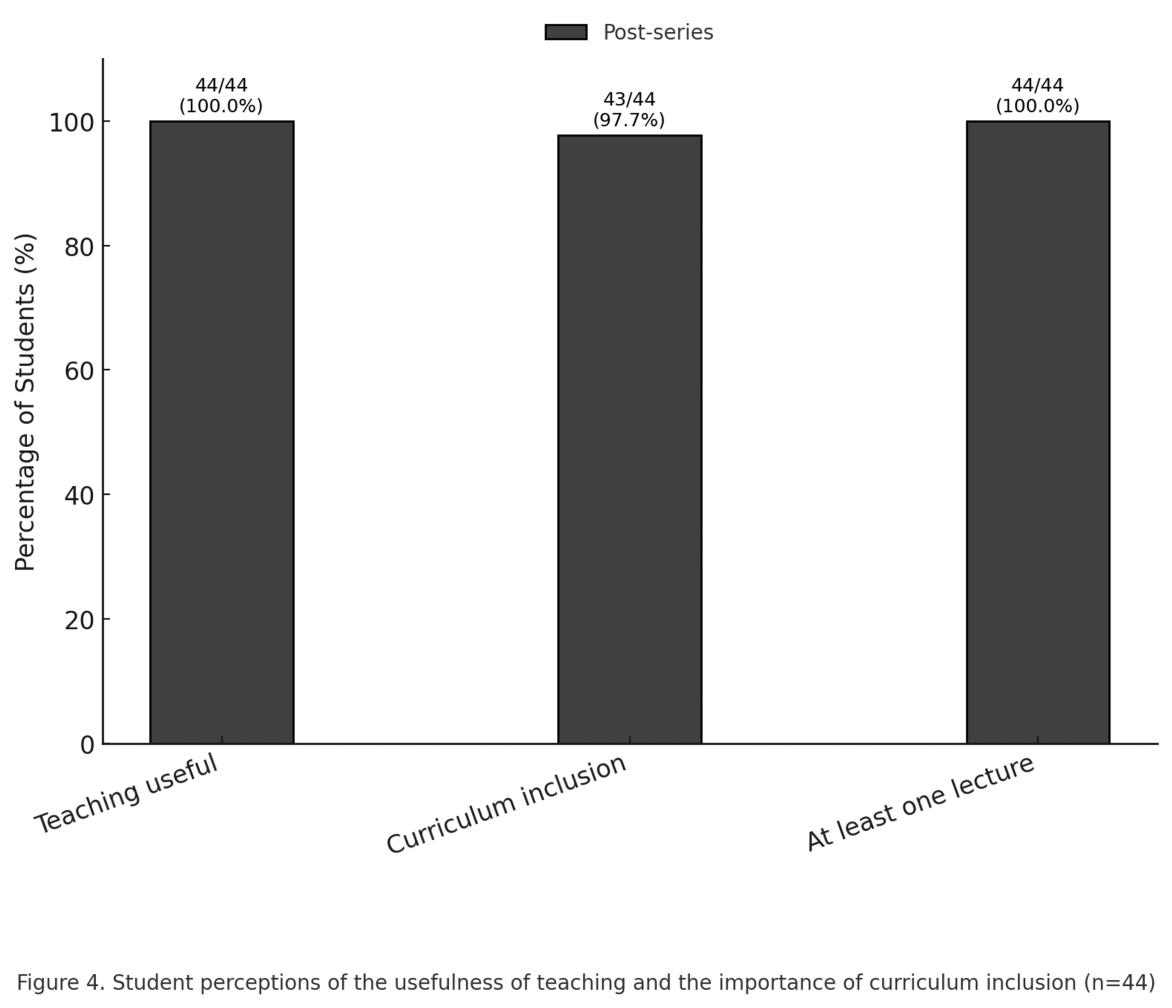
Change in student perception of the importance of skin of colour (SoC) for clinical practice (n = 44)

All students rated the teaching as useful (100%, 95% CI 92.0-100.0). Almost all (98%, 43/44) agreed that SoC should be included in the undergraduate curriculum (95% CI 88.2-99.6). In addition, every student (100%, 44/44) agreed that at least one lecture on diverse skin presentations should form part of medical training (95% CI 92.0-100.0). The data are presented in Table 3 and illustrated in Figure 4.

**Table 3:** Participant perceptions regarding skin of colour (SoC) teaching (n = 44)

Survey item	Agree/yes n (%)	Disagree/no n (%)
Previously received formal skin of colour dermatology teaching	5 (11%)	39 (88%)
Believe skin of colour dermatology is important for future practice	43 (98%)	1 (2%)
Support inclusion of skin of colour dermatology in medical school curricula	43 (98%)	1 (2%)

## Discussion

This study shows that a structured, student-led online teaching series can improve medical students' perceived confidence when faced with dermatological conditions in SoC. Before the intervention, students reported limited exposure and lower confidence compared with GenDerm. After the lectures, confidence levels shifted significantly in both areas, with no students remaining in the not at all confident categories. Wilcoxon signed-rank tests confirmed these changes were significant, with large effect sizes for GenDerm and very large effect sizes for SoC. To reiterate, the goal of the study was to bring confidence with SoC more in line with confidence in GenDerm and, hence, to bridge the gap.

Figure 2 and Figure 3 demonstrate similar distributions, with upward shifts in confidence scores that mirror one another. These visual patterns are consistent with the statistical analysis, supporting our hypothesis that the gap between GenDerm and SoC confidence can be bridged. This finding is consistent with recent reports highlighting both the scarcity of formal SoC teaching in medical curricula [5,7-9] and the value of focused interventions in improving student confidence [10-12]. The consensus among our participants in favour of integrating SoC into the undergraduate curriculum further emphasises the need for systemic change. Addressing these gaps is important not only for enhancing student confidence but also for supporting the development of equitable patient care in the longer term [1-4]. The online delivery format offers the additional advantages of being cost-effective, scalable and easily replicable. 

Strengths and limitations 

The study has several strengths. It represents a multi-institutional collaboration, was designed and led by medical students with expert input. The combination of quantitative data on confidence levels and qualitative feedback on perceptions provides both statistical and contextual evidence of educational value. 

Our study did have limitations to note. The survey instrument, whilst not formally psychometrically validated, underwent rigorous content validation by clinical experts with educational expertise. Likert scales are often used in medical education analysis with good effect [14]. The outcomes, therefore, reflect Kirkpatrick levels 1 and 2 (reaction and self-perceived learning) [15], rather than demonstrating changes in behaviour (level 3) or patient outcomes (level 4). The sample size was modest, and voluntary participation introduces the possibility of selection bias. 

Whilst self-reported confidence improved, this does not necessarily reflect objective diagnostic skill. Future work should include validated knowledge tests or Objective Structured Clinical Examination (OSCE)-style assessments, as these are needed to validate these findings and explore the potential impact on clinical performance.

## Conclusions

Medical students reported lower confidence in diagnosing SoC compared with GenDerm. Our nine-lecture series improved self-reported confidence and received near-universal support for curricular inclusion. Incorporating structured SoC teaching into undergraduate education may help to reduce inequalities in dermatological care, although further studies using validated assessments are needed to confirm the impact on clinical practice.

## Appendices

The surveys were originally created and administered using Microsoft Forms. For the purposes of publication and replicability, the questions are included in this document.

Explanation of terms

Participants were given the following explanation for terms:

• General dermatology (GenDerm): refers to skin conditions in lighter skin tones.

• Skin of colour dermatology (SoC): refers to the same conditions as seen in patients with darker skin tones.

Pre-lecture survey

1. Are you a student or healthcare professional?

☐ Student

☐ Healthcare professional

2. Which university or hospital do you attend?

 [Free text response]

3. Have you previously received teaching on SoC dermatology?

☐ Yes

☐ No

4. How confident do you feel with general dermatology conditions?

☐ 1 = Not at all confident

☐ 2 = Slightly confident

☐ 3 = Somewhat confident

☐ 4 = Quite confident

☐ 5 = Extremely confident

5. How confident do you feel with SoC dermatology conditions?

☐ 1 = Not at all confident

☐ 2 = Slightly confident

☐ 3 = Somewhat confident

☐ 4 = Quite confident

☐ 5 = Extremely confident

6. Do you feel more, less, or the same level of confidence diagnosing conditions in lighter (white) versus darker (SoC) skin?

☐ More confident in White skin

☐ Same confidence in both

☐ More confident in SoC skin

7. Do you think medical schools should have at least one lecture on diverse skin presentations?

☐ Yes

☐ No

Post-lecture survey

1. Are you a student or healthcare professional?

☐ Student

☐ Healthcare professional

2. Which university or hospital do you attend?

[Free text response]

3. Was this lecture series the first teaching session you had on dermatology for patients of colour?

☐ Yes

☐ No

4. Did you find this method of teaching useful?

☐ Yes

☐ No

5. How confident do you feel with general dermatology conditions?

☐ 1 = Not at all confident

☐ 2 = Slightly confident

☐ 3 = Somewhat confident

☐ 4 = Quite confident

☐ 5 = Extremely confident

6. How confident do you feel with SoC dermatology conditions?

☐ 1 = Not at all confident

☐ 2 = Slightly confident

☐ 3 = Somewhat confident

☐ 4 = Quite confident

☐ 5 = Extremely confident

7. How much do you agree with the following statement: ‘Learning about dermatology in patients of colour is important for your future clinical practice.’

☐ 1 = Strongly disagree

☐ 2 = Disagree

☐ 3 = Neutral

☐ 4 = Agree

☐ 5 = Strongly agree

8. Do you think medical schools should have lectures on SoC dermatology in their curriculum?

☐ Yes

☐ No
